# Editorial: Molecular mechanisms in lethal states of prostate cancer

**DOI:** 10.3389/fonc.2024.1475178

**Published:** 2024-08-20

**Authors:** Daniel M. Kim, Yung Lyou, Leigh Ellis, Edwin Posadas, Neil Bhowmick, Jun Gong

**Affiliations:** ^1^ Samuel Oschin Comprehensive Cancer Institute, Cedars-Sinai Medical Center, Los Angeles, CA, United States; ^2^ Department of Oncology, Providence St. Jude Medical Center, Fullerton, CA, United States; ^3^ Department of Surgery, Center for Prostate Disease Research, Murtha Cancer Center Research Program, Uniformed Services University of the Health Sciences and the Walter Reed National Military Medical Center, The Henry M. Jackson Foundation for the Advancement of Military Medicine, Inc., Bethesda, MD, United States

**Keywords:** castration-resistant prostate cancer (CRPC), castration-sensitive prostate cancer, microRNA, androgen signaling pathway, bony metastasis, novel hormonal therapy

In 2024, prostate cancer is the most commonly diagnosed cancer and second leading cause of cancer-related mortality in American men (1). However, in patients with localized or regional prostate cancer, the 5-year relative survival is near 100% (2). The excellent prognosis in localized or regional prostate cancer is due to its frequently indolent activity and the effectiveness of androgen deprivation therapy (ADT), surgery, and radiotherapy early in the disease course (3). Death due to prostate cancer results from progression to castration resistance and metastasis (4). The mechanisms that cause an indolent and treatable disease to a lethal cancer is an ongoing area of research. In this editorial, we will highlight a few notable molecular drivers of lethality. Our focus will be on aberrations in the androgen receptor pathway, microRNA, and bony microenvironment, as highlighted by a series of studies published under the Research Topic “Molecular Mechanisms in Lethal States of Prostate Cancer.” We will place findings from these recent studies in the broader context of mechanisms that cause resistance or metastasis, while touching upon a few additional topics of emerging interest in this area. In doing so, we hope to identify potential targets of therapy that can improve survival of men with lethal prostate cancer.

The androgen signaling pathway plays a critical role in prostate carcinogenesis and aggressiveness (5). Targeting this pathway via ADT is a mainstay and effective treatment in almost all stages of prostate cancer. Whether localized disease or *de novo* metastatic disease, evolution to more aggressive disease states is characterized by progression from castration-sensitive to castration-resistant prostate cancer (CRPC) (6). Treatment for metastatic prostate cancer in the modern era is characterized by ADT intensification (7). Recent approvals in the treatment of metastatic castration-sensitive prostate cancer (mCSPC) include the addition of docetaxel (8, 9) or novel hormonal therapies to ADT in the first-line setting (10–14). In a subset of high-volume mCSPC patients, triplet therapies with ADT, docetaxel, and a NHT have been established (15, 16). Despite these recent breakthroughs with ADT intensification, progression to castration-resistance is nearly universal whereby mechanisms of castration-resistance has undergone extensive research (17–19). Although metastatic CRPC (mCRPC) has recently seen breakthroughs in targeted and non-targeted systemic therapies, mCRPC often represents the lethal end-stage of prostate cancer (7). There remains a high unmet need to understand molecular pathways involved in the progression of prostate cancer to lethal states with the hopes of identifying novel biomarkers or therapeutic targets to improve outcomes in patients with lethal forms of prostate cancer.

White III et al. recently proposed Src kinase as a candidate. The group found that Src kinase, a non-receptor tyrosine kinase, regulated both normal AR and AR splice variants. They also speculated that Src kinase was involved with NHT resistance via upregulation of non-AR steroid receptors, resulting in the bypassing of AR signaling for growth. In their *in vitro* study, inhibition of Src kinase with saracatinib resulted in ablation of the phosphorylation of AR Y^534,^in cells expressing both normal AR or AR splice variants. As a result, AR expression and activity was decreased regardless of AR status. They also revealed a strong synergism with saracatinib and enzalutamide which resulted in a greater reduction of AR expression and activity and increased apoptosis. The proposed mechanism was that saracatinib would inhibit AR expression, AR activity, and prevent the upregulation of non-AR steroid receptors induced by enzalutamide alone. Thus, Src kinase may be a therapeutic target. We anticipate future *in vivo* and human research to see if NHT and Src kinase inhibition would be a viable combination therapy.

MicroRNAs are non-coding, regulatory RNAs that have important roles in many cancers, including prostate cancer (20). They can act as oncogenes, tumor suppressors, and even as mediators of lineage plasticity (21–23). With dysregulation, these microRNAs can promote carcinogenesis and metastasis. Duca et al. investigated this in mice models and prostate cancer genetic databases. They discovered that in mice injected with prostate cancer cells, high-fat diets (versus normal diets) downregulated miR-133a-3p and miR-1a-3p, resulting in increased tumor growth. A similar downregulation of miR-133a-3p and miR-1a-3p was observed in human prostate cancer samples compared to normal prostate tissue. Thus, this study suggests the role of miR-133a-3p and miR-1a-3p as tumor suppressors in prostate cancer. Additionally, miR-133a-3p was found in lower levels in patients with metastasis compared to no metastasis, suggesting miR-133a-3p as a protective factor against metastasis. This finding reveals an interesting role of microRNA as mediators of metastasis. Indeed, in a study by Cassidy et al., they demonstrated *in vitro* and *in vivo* that overexpression of miR-379 played a protective role against prostate cancer metastasis. They also saw that in human tissue, lower miR-379 levels were observed in primary tumor samples and metastatic bone samples compared to benign tissue. Two mechanisms were suggested. MiR-379 increased cell-cell adhesion, preventing cancer cells from breaking off the primary tumor and therefore decreasing systemic spread of cancer. And miR-379 decreased cell-bone adhesion which would prevent cancer cells from seeding in the bone, the most common metastatic site. Therefore, when miR-379 is downregulated, there is a more favorable environment for metastasis. Given these findings, further research is necessary to see how miRNA can regulate the progression of prostate cancer into a metastatic and lethal disease.

Since the most frequent metastatic site for prostate cancer is the bone, the bony microenvironment has been a highly studied area of interest (24, 25). In the bone, chemokines play a major role in promoting prostate cancer metastasis and growth, which Johnson and Cook outlines in their review. These chemokines include CXCL8, CXCL12/CXCR4, CCL2, CCL5/CCR5/CCR4, CXCL13, CXCL16, and CX3CL1/CX3CR1. From their review, a few notable findings have emerged. CXCL8, CXCL12, CXCL13, CXCL16, CCL2, CCL5, and CX3CL1 are involved with cancer cell migration and invasion. CXCL8, CXCL16, and CCL2 are involved with angiogenesis. CXCL8, CXCL12, CXCL13, CCL2, CCL5, and CX3CL1 are involved with cancer proliferation. CXCL8, CXCL12, and CCL2 are involved with osteoclastogenesis and osteolysis. And CXCL8 and CXCL16 are involved with chemoresistance. As the function of these chemokines in prostate cancer are being discovered, their potential clinical utility becomes unveiled. For example, CCL5 and CXCL8 were elevated in patients with metastatic and aggressive prostate cancer and can potentially function as biomarkers. Therapies against CCR5, CCR4, and CXCR4 are already developed and can potentially block pro-tumorigenic signals and metastasis. CCL2 inhibition showed promising pre-clinical potential, but a clinical trial failed to show anti-tumor activity with single-agent CCL2 inhibition (26). This shows that as promising as pre-clinical studies might be, they must be validated in the clinical setting. As such, further work into the clinical application of these chemokines must be pursued.

Beyond these recent studies, our group and others have demonstrated that glutamine metabolism plays a role in evolution of prostate cancer to more therapy-resistant and aggressive states of disease (27). Here, increased glutamine dependency or “addiction” has been associated with prostate cancer progression from castration-sensitive to castration-resistant states and neuroendocrine differentiation across pre-clinical prostate cancer models (27). Combination strategies to target glutamine metabolism in prostate cancer are in active investigation. We have also shown that the systemic antagonism of BMP/CD105 signaling can support the re-sensitization of castration-resistant prostate cancer to androgen receptor signaling inhibitors (ARSIs) through downregulation of AR-V7 (28). We are currently conducting a phase II randomized trial of men with mCRPC progressing on previous ARSI therapy to receive apalutamide with or without carotuximab (anti-CD105 monoclonal antibody) (29). EZH2 inhibition has also shown promise in pre-clinical prostate cancer models through the potential to enhance immune response through activation of the dsRNA-STING-interferon stress axis (30).

Although early and localized cases of prostate cancer have optimistic outcomes, metastatic and castration-resistant prostate cancer remains lethal. Aberrations occurring in the above pathways all play an interconnected role that results in metastatic, castration-resistant prostate cancer. However, these findings represent some of the many recent and ongoing efforts to identify novel mechanistic and therapeutic targets to improve our care of patients with lethal prostate cancer states. The pre-clinical data for these mechanisms are hopeful. But they must be validated in the clinical context. Given this, further research is still needed to implement these results to the bedside. We hope that as these findings become clinically useful, we will be able to improve the survival of men suffering from lethal prostate cancer.
